# Kallmann Syndrome: Eugenics and the Man behind the Eponym

**DOI:** 10.5041/RMMJ.10242

**Published:** 2016-04-19

**Authors:** Carlos A. Benbassat

**Affiliations:** 1 Endocrine Institute, Assaf Harofeh Medical Center, Zerifin, Israel; 2 Sackler Faculty of Medicine, Tel Aviv University, Tel Aviv, Israel

**Keywords:** Eugenics, Kallmann syndrome, Nazi medicine, racial hygiene

## Abstract

Kallmann syndrome is named after Franz Joseph Kallmann, a German-born psychiatrist who described in 1944 twelve subjects from three families who presented with a syndrome of missed puberty, anosmia, and color blindness. Yet, several other eponyms for the same syndrome can be found in the literature. Despite the fact that Kallmann syndrome is the most recognized eponym, very little is known about the man for whom the syndrome is named. A biographical note on Franz Joseph Kallmann and his historical context is presented.

## INTRODUCTION

Kallmann syndrome is a congenital disorder of the hypothalamus, characterized by reduced pituitary gonadotropic activity resulting in hypogonadism with associated anosmia. In 1856, Aureliano Maestre de San Juan described the autopsy findings in a 40-year-old man with absent olfactory lobes, small testicles, small penis, and no pubic hair.^1^ Several years later, Richard L. Heschl reported similar findings in a 45-year-old man. In 1914, Franz Weidenreich conducted a post-mortem study of 10 individuals with anosmia, 3 of whom had hypogonadism, and suggested that these problems might be an associated syndrome.^2^ It was not until 1944, however, that Kallmann and colleagues drew attention to the genetic etiology of the disorder in a study of 12 subjects from three families who presented with a syndrome of missed puberty, anosmia, and color blindness. Two of them were also mentally retarded.^3^ Ten years later, De Morsier documented 14 affected persons and confirmed that the anosmia was a consequence of agenesis of the olfactory lobes.^4^ When and how the disease was eponymously termed Kallmann syndrome remains unclear, and some synonymous names can be found in the literature. In this article, we describe the medical history and background of the man behind the eponym.

## FRANZ KALLMANN: THE EARLY YEARS

Franz Joseph Kallmann was born in 1897 in Neumarkt, Silesia, Germany, the son of Marie and Bruno Kallmann. Bruno was a physician who converted from Judaism to Christianity.^5^ After completing his military service, Franz Joseph received an MD degree from the University of Breslau in 1919 and underwent further training in psychiatry with Karl Bonhoffer and Hans Gerhard Creutzfeld in Berlin. Early in his career, Kallmann became interested in theories of genetic factors in schizophrenia, and between 1929 and 1935 he was granted several fellowships at the Psychiatric Research Institute in Munich, in the department headed by the Swiss psychiatrist Ernst Rudin.^5^ Rudin was the brother-in-law of Alfred Ploetz, with whom he founded the Munich Society for Racial Hygiene.^6^ In an article published in 1903, “Alcohol in the Life Process of the Race,” Rudin wrote (translated by Weber)^7^ that to obtain “biologically fit members of the race” it was necessary not only to have “maximum propagation of those who are healthy, robust and … ethically superior” but also to exclude “the weak, ill, unfit and morally reprehensible from reproduction by artificial selection, … by instruction and by private and government force.” In 1916, Rudin published his pivotal study on 701 families in which he sought to demonstrate the heritable characteristics of schizophrenia.^7^,^8^ Rudin was appointed president of the International Federation of Eugenics Organizations in 1932 and, from 1935 to 1945, served as president of the Association of German Neurologists and Psychiatrists.^7^ Franz Kallmann joined Rudin when he was the director of the Genealogical-Demographic Department at the Psychiatric Research Institute in Munich.

## GERMANY IN THE EARLY TWENTIETH CENTURY: FROM EUGENICS TO NAZI MEDICINE

The eugenics movement emerged in Europe and North America at the end of the nineteenth century. It was rooted in the biological determinist ideas of Social Darwinists. The term eugenics is attributed to Sir Francis Galton, who was Darwin’s first cousin. Galton studied the upper classes of Britain and concluded that their social positions were a product of their superior genetic background.^8^ Early proponents of eugenics believed in the genetic superiority of Nordic, Germanic, and Anglo-Saxon peoples. In 1920, the psychiatrist Alfred Hoche along with Karl Binding published the book, *Release and Destruction of Lives Not Worth Living*, a prelude to the concept of the systematic elimination of patients with mental disorders.^8^ At the end of World War I, the Weimar Republic looked to popular eugenics theories in an attempt to improve the health and physical well-being of the populace. In 1932, inspired by Harry Laughlin’s *Eugenical Sterilization in the United States* and other American writings, the Weimar government drafted a plan for the sterilizations of individuals with hereditary diseases.^9^ The next year, the National-Socialist (Nazi) party took control of Germany and, on July 14, 1933, issued the Law for the Prevention of Progeny with Hereditary Diseases. Ernst Rudin, Kallmann’s mentor at that time, co-authored the official commentary and guidelines for its implementation.^7^,^10^ The law stipulated that 200 Genetic Health Courts be established nationwide where teams of lawyers and doctors would review medical records and select individuals with heritable diseases (defined as congenital feeble-mindedness, schizophrenia, manic depression, hereditary epilepsy, Huntington’s chorea, hereditary blindness, hereditary deafness, and serious physical deformities) for voluntary or forced sterilization.^9^ Two years later, Franz Kallmann gave a presentation at the International Congress for Population Science in Berlin hosted by the Interior Ministry of Hitler’s government. In his speech (translated by Muller-Hill^11^), Kallmann proposed that the program be extended to relatives of individuals with schizophrenia in order to identify also non-affected carriers (that is, those with minor anomalies) for compulsory sterilization.^11^,^12^ In the six years before World War II, Nazi doctors sterilized some 400,000 people, mostly German citizens living in asylums.^9^,^11^ Sterilization gave way to killing with the approval of the *Aktion T-4* program in 1939. Named after the location of its headquarters in Berlin (4 Tiergarten Street), the program was run by 50 volunteer physicians, authorizing specific doctors and officials to carry out mercy deaths/euthanasia of defective individuals, as recommended by physicians and psychiatrists throughout Germany (for detailed list see Lindert et al.^13^). Methods of killing included starvation, morphine injections, tablets, and gassing.^14^ Eventually, church groups and the general public raised objections, and the program was officially halted in August 1941. By then, almost 70,000 people had been killed. However, unofficially, the killing continued to the end of the war. During the period from 1934 to 1945, an estimated one-third (132,000) of the 400,000 sterilized individuals had a diagnosis of schizophrenia. In the same period an estimated 200,000–275,000 psychiatric patients were killed, of whom 100,000–137,500 were patients with schizophrenia.^9^ Altogether and according to Torrey et al.^9^ some 250,000 schizophrenics were killed or sterilized, representing more than 70% of the estimated schizophrenic population in Germany during that period. One could say that the Nazi party took Kallmann’s recommendations one step forward. In 1942, Rudin published an article summarizing the last 10 years of the National Socialist State in which he stated (translated by Muller-Hill^11^): “It will always remain the historic achievement of Adolf Hitler … to take the firsts steps towards such brilliant race-hygiene … the fight against parasitic alien races as the Jews and the Gypsies … and preventing the breeding of those with hereditary disease.”

## FRANZ KALLMANN: THE POST-WAR YEARS

Dr Franz Kallmann was half Jewish, and his wife Helly was Lutheran. In 1936, his sister-in-law, a devoted Nazi functionary, suggested to her sister that either she divorce Dr Kallmann or leave the country.^12^ Therefore, in 1936, Kallmann interrupted his study of hereditary degeneracy and immigrated with his wife to America. He subsequently established the Medical Genetics Department of the New York State Psychiatric Institute (NYSPI), which was at the time affiliated with Columbia University. Two years after his arrival to the States, Kallmann completed his first major work, *The Genetics of Schizophrenia*, published simultaneously in the USA and Germany, based on data derived from 13,851 relatives of 1,087 German patients admitted to a Berlin hospital over a 10-year period. The results showed that first-degree family members of patients with schizophrenia have a significantly higher risk of the disease (16.4% in children, 11.5% in siblings) than the general population (0.85%), suggesting that schizophrenia has a hereditary component. However, cultural transmission could not be completely ruled out.^15^ In the acknowledgments, Kallmann thanked his long-time mentor, Dr Ernst Rudin.^12^ The book came out just one year before the Aktion T-4 unit begun murdering patients with mental disorders and other “defects.”

Kallmann was also sought to identify a genetic basis for homosexuality, another target of the Nazi racial hygiene program. In an article published in 1952,^16^ he wrote: “Taking notice of this generally unsatisfactory state of information about the genetic aspects of adult homosexual behavior … our investigative program was planned as a concentric and strategically coordinated attack from different directions.” He concluded: “In any case, the need of additional work in relation to the genetic aspects of homosexuality cannot possibly be questioned. The urgency of such work is undeniable as long as this aberrant type of behavior continues to be an inexhaustible source of unhappiness, discontentment, and a distorted sense of human values.” This study supported the earlier report of Theobald Lang, which had been criticized because of statistical inadequacy.^16^ Lang, a German psychiatrist and medical assistant to Dr Rudin in the Munich Institute, had been assigned by Dr Rudin to deliver the German data on schizophrenic patients and relatives to Dr Kallmann in America for his book.^12^ He sympathized with the National Socialism movement, which he found to be a “… political expression of our biological knowledge.”^14^ It was only after several trial proceedings following WWII that Lang was “denazified.”

## EUGENICS IN AMERICAN ACADEMIA IN THE EARLY TWENTIETH CENTURY

In 1906, the American Breeder’s Association became the first eugenics body established in the US, under the directorship of the biologist Charles B. Davenport. The association was formed specifically to investigate heredity in the human race, with an emphasis on the value of superior blood and the menace to society of inferior blood.^17^ The Eugenics Record Office was founded in 1911 in Cold Spring Harbor, New York, also by Davenport, and evolved into one of the leading organizations in the American eugenics movement. In 1916, American eugenics leader Madison Grant published his book, *The Passing of the Great Race*, which earned the admiration of Hitler, who quoted it in *Mein Kampf*.^9^ The first state-sponsored sterilization legislation in the US was enacted in 1907 in Indiana, followed two years later by Washington and California. Harry Laughlin’s proposal *Eugenical Sterilization in the United States*, the “model for law” for the Nazi program of compulsory sterilization, served as the model for the American program as well. Subjects considered for sterilization included the feeble-minded, the insane, criminals, epileptics, alcoholics, blind persons, deaf persons, deformed persons, and indigent persons. Following Laughlin’s proposal, an additional 18 states passed sterilization laws. In terms of numbers, the state of California was at the vanguard of the American eugenics movement, accounting for one-third of the 60,000 sterilizations performed nationwide from 1909 to the 1960s. North Carolina had the longest-lasting eugenics program, which was operative until 1977.^8^,^17^

At the beginning of the twentieth century, around the time Kallmann immigrated, eugenics was widely accepted in the US academic community. In 1928, a total of 376 university courses, attended by some 20,000 students, included eugenics in the curriculum.^18^ During the 1930s, America’s most prominent universities, including Harvard, Yale, and Columbia, welcomed Nazi leaders to their campuses. In December 1933, Columbia’s Nicholas Murray Butler hosted a reception for the then German ambassador, Hans Luther. Although journalists had been reporting for years that the Hitler regime was very oppressive of some minorities, Butler dismissed criticism over the invitation and praised the Nazi ambassador as “the diplomatic representative of a friendly people.”^18^ One year later, Harvard University in association with the student newspaper, the *Crimson,* warmly welcomed Ernst Hanfstaengl, the Nazi party foreign press chief during a visit from Germany; Hitler had taken refuge in Hanfstaengl’s villa outside Munich after the 1923 Beer Hall Putsch. The acceptance of eugenics among American faculties lasted into the 1940s. Indeed, in 1942, the *American Journal of Psychiatry* published a proposal by Foster Kennedy from Cornell University to kill retarded children aged 5 or older “… who should never have been born.”^19^,^20^

## FRANZ KALLMANN: THE LATER YEARS

This was the scenario in Europe and America when Dr Kallmann moved from the Rockefeller- and Nazi-funded Psychiatry Research Institute in Munich^7^,^12^ to the Columbia University-affiliated NYSPI. At the end of the war, Kallmann certified that Ernst Rudin was only a nominal member of the Nazi party, helping him to obtain complete exoneration. A letter he wrote at Rudin’s request read as follows (quoted from Gottesman et al.^6^): “He is no criminal, of course, and he certainly deserved a better fate at the end of a long life which was devoted to scientific progress and the betterment of mankind as he understood it. If he had to be penalized, he received sufficient punishment through the complete collapse of his scientific dream world and through the irreparable damage to his reputation as a scientific worker.”

In the following years, Dr Kallmann served at the forefront of genetic psychiatry. In 1944, he published a description of several families in which a majority of the members had all or some of the symptoms of missing puberty, anosmia, and color blindness, drawing attention to the genetic etiology of the disorder that was later named Kallmann syndrome.^4^ In 1948, he became one of the founders of the American Society of Human Genetics, and in 1954 he was appointed full professor at Columbia University. From 1952 to 1965, he served on the Board of Directors of the American Eugenics Society. Dr Kallmann died in 1965 at age 68 in New York.^21^

## CONCLUSION

As editor of a local endocrine newsletter, I became acquainted with this piece of history while searching for a historical note to be included. From Harvey Cushing to Fuller Albright, the endocrine literature is full of biographic notes; however, Gershon’s is the only biographic article on Franz Kallmann that I could find in medical journals.^5^ Although Kallmann syndrome is often encountered by endocrinologists in daily practice, very few know the story of the man behind the eponym. Franz Kallmann was not a Nazi party member and was not involved in the atrocities committed by his contemporary German colleagues; however, he has been criticized in several articles, mostly in the psychiatric arena, for his eugenics views. Some authors have profiled him as a Nazi victim,^5^,^22^ and others as a victimizer.^9^,^12^,^23^ Though his views were probably tempered over the years, the fact that his work both abroad and in America emerged from the epicenter of Nazi medicine raises concerns about the ethical implications of the eponymous distinction bestowed upon him. It took almost 70 years for the German Psychiatry Association to apologize for their complicity with Nazi killings,^24^ and it was not until a few years ago that some states in America began considering compensation for the victims of forced sterilization.^25^ As Strous and Edelman^22^ commented in their paper “Eponyms and the Nazi era,” there is a time to remember and a time to change. We dare not forget that forced sterilizations and euthanasia in the first half of the last century were “scientifically” justified in order to improve the human race. In the current era of commercially available human genome sequencing and genetic counseling, the question of who deserves to live and what lives are worthy of living should never be raised again.
